# Revert to the original: time to re-establish delayed umbilical cord clamping as the standard approach for preterm neonates

**DOI:** 10.1186/s40748-018-0081-5

**Published:** 2018-07-04

**Authors:** Ryan M. McAdams, Carl H. Backes, Omid Fathi, David J. R. Hutchon

**Affiliations:** 1 Department of Pediatrics, Division of Neonatology, University of Wisconsin School of Medicine and Public Health, 1010 Mound St., Rm 414, Madison, WI 53715 USA; 2 Department of Pediatrics, The Center for Perinatal Research and The Heart Center, Nationwide Children’s Hospital, Columbus, OH USA; 3 Obstetrics and Gynaecology, Darlington Memorial Hospital, Darlington, UK

## Abstract

Delayed cord clamping, the common term used to denote placental-to-newborn transfusion at birth, is a practice now endorsed by the major governing bodies affiliated with maternal-newborn care. Despite considerable evidence, delayed cord clamping, not early cord clamping, continues to be viewed as the “experimental” intervention category when discussed in research studies. We provide a brief overview of placental-to-newborn transfusion in relation to birth transitional physiology and discuss areas where we may need to modify our interpretation of “normal” vital signs and laboratory values as delayed cord clamping becomes standardized. We also assert that delayed cord clamping should now be viewed as the standard of care approach, especially given that multiple randomized controlled trials have revealed that early cord clamping, which lacks evidence-based support, is associated with a greater risk for morbidity and mortality than delayed cord clamping.

**Electronic supplementary material:**

The online version of this article (10.1186/s40748-018-0081-5) contains supplementary material, which is available to authorized users.

*“What’s in a name? that which we call a rose/By any other name would smell as sweet.”*

― William Shakespeare, Romeo and Juliet.

Similar to Shakespeare’s poetic use of dramatic structure, shifting between comedy and tragedy to increase tension, practices in neonatal medicine have also switched back and forth. Sentiments toward oxygen use in neonates have alternated between good and bad; [1] despite a wealth of research, the debate on the optimal amount of supplemental oxygen exposure in neonates is still unresolved. However, supplemental oxygen remains necessary to keep fragile babies alive, but this benefit is also associated with morbidities, such as chronic lung disease and retinopathy of prematurity. Although many questions remain unanswered regarding insufficient versus excessive oxygen delivery, research and scrutinized evidence have improved our understanding of how oxygen impacts neonates. Another area of debate and alternating practice deals with placental transfusion. Delayed cord clamping, the common term used to denote placental-to-newborn transfusion at birth, is a practice now endorsed by the major governing bodies affiliated with maternal-newborn care [2–5]. While most of the estimated 108 billion people who have existed on earth [6] likely had delayed cord clamping performed at birth, early or immediate cord clamping became the standard in the 1900’s, a practice introduced and adopted without evidence. Fast forward to the current day and you will find substantial evidence supporting the original, natural approach of delayed cord clamping at birth. However, despite considerable evidence, delayed cord clamping, not early cord clamping, remains stuck in the “experimental” intervention category when discussed in research studies.

We provide a brief overview of placental-to-newborn transfusion in relation to transitional physiology at birth and discuss areas where we may need to modify our interpretation of “normal” vital signs and laboratory values as delayed cord clamping becomes standardized. We also assert that delayed cord clamping should now be viewed as the standard of care approach, especially given that multiple randomized controlled trials have revealed that early cord clamping, which lacks evidence-based support, is associated with a greater risk for morbidity and mortality than delayed cord clamping [7, 8].

## Normal physiological transition after birth

The respiratory and cardiovascular changes after birth are coordinated and largely completed within a few minutes. Chest expansion results in biochemical processes that lead to alveolar inflation with air and pulmonary vasodilation associated with a fourfold increase in pulmonary vascular blood flow [9]. Air oxygen content further increases pulmonary vascular vasodilation enabling the pulmonary vascular bed to accommodate all the right ventricular output. Increased blood oxygen content activates mitochondrial oxygen sensors in the ductus arteriosus tissue, generating H_2_O_2,_ which inhibits ductal smooth muscle cell voltage-dependent potassium channels leading to intracellular calcium influx and overload, a process that promotes ductal vasoconstriction [10]. As pulmonary blood flow increases, neonatal-to-placental circulation decreases due to umbilical arterial vasoconstriction prompted by increased oxygen tension and bradykinins released from the neonatal lungs. Umbilical venous flow results in a net volume of blood (24–32 ml/kg of birth weight) moving from the placental to the term neonatal circulatory bed [11]. This blood volume, designated the placental transfusion, helps expand the pulmonary vascular bed and maintain the normal circulating volume of the neonate. In preterm neonates, information on net blood volumes related to umbilical venous flow during placental-to-neonatal transfusion is lacking. However, a study by Aladangady et al., which used a biotin-labeled, autologous red blood cell dilution method to examine placental blood transfer in 46 neonates ranging from 24 to 32 weeks’ gestation assigned randomly to either delayed cord clamping (90 s; median) or early cord clamping (10 s; median) provides insight into placental transfusion volumes in preterm neonates [12]. This study demonstrated mean blood volume in the delayed cord clamping group (74.4 mL/kg) was significantly greater than that in the early cord clamping group (62.7 mL/kg), a difference of 11.7 ml/kg.

The placental-to-neonatal circulation usually ceases between 2 and 5 min after delivery, having contributed up to 30% of the neonate’s total potential circulating blood volume at birth [11]. With absent placental-to-neonatal blood flow, levels of vasodilatory prostaglandins produced by placental cyclooxygenase enzymes diminish, further inducing ductus arteriosus constriction. The increased pulmonary blood flow also leads to increased prostaglandin clearance by the lung, which further contributes to ductal constriction [13].

The majority of our knowledge regarding transitional neonatal circulation has been derived from neonates who have tolerated early cord clamping without any apparent effect on their health [14] or more recently came from babies delivered by caesarean section [15]. The current observations of ductus arteriosus physiology, with the ductus remaining functionally open for at least 12 h after birth [14] and a persistently patent ductus arteriosus present in 0.05% of healthy term neonates [13] are based on studies of newborns who had early cord clamping. How delayed cord clamping impacts ductal patency patterns in term and preterm neonates remains unclear, but may be different than the pattern of ductal patency and closure associated with early cord clamping.

In the transitioning newborn, ensuring adequate end-organ oxygen delivery is important to prevent hypoxic injury. Systemic oxygen delivery is dependent on the product of cardiac output and arterial oxygen content. Cardiac output, the product of heart rate and stroke volume, is dependent on preload. Venous return increases preload and therefore, delayed cord clamping, which promotes placental-to-neonatal transfusion that augments venous blood flow to the right heart, should improve cardiac output and oxygen delivery. In addition, since blood oxygen carrying capacity is mainly determined by hemoglobin (Hb) concentration and Hb-oxygen saturation, the increased Hb level resulting from delayed cord clamping also should increase arterial oxygen content. By clamping the cord early, both preload and Hb levels may be decreased, which could diminish systemic oxygen delivery and potentially contribute to end-organ injury.

Under hypoxic conditions, oxygen extraction is critically dependent on blood flow [16], which should raise questions about how decreased flow due to early cord clamping could contribute to adverse outcomes in compromised premature neonates. Delayed cord clamping may not only augment systemic oxygen delivery as described above, but may also promote better blood flow and therefore, facilitate increased oxygen extraction in the setting of hypoxia. If this improved blood flow results in better cerebral perfusion and oxygenation, these factors may at least partially explain why delayed cord clamping results in decreased intraventricular hemorrhage [7, 17]. An additional point to contemplate is how cord management practices may affect tissue oxygen delivery by altering the amount of fetal Hb. Since the arterial partial pressure of oxygen when Hb is 50% saturated with oxygen (i.e., p50) is lower with fetal Hb compared to adult Hb, neonates with more fetal Hb will have better oxygen extraction and delivery [16]. This difference in fetal versus adult Hb concentrations warrants consideration since premature neonates exposed to early cord clamping receive more blood transfusions than neonates who had delayed cord clamping [17], and these neonates are transfused with packed red blood cells containing adult Hb. Neonates who had delayed cord clamping, who do not require as many blood transfusions, maintain a higher percentage of fetal Hb, at least in the short-term, which may be important in how they are affected by hypoxic stressors (e.g., sepsis, respiratory failure, apneic episodes, and hypoxic ischemic encephalopathy). Ignoring the possible role of cord clamping practices on these variables dismisses physiologic principles of tissue oxygenation and hinders insight into modifiable mechanisms, which may improve or worsen outcomes in premature neonates.

During the critical period of transition at birth, the hemodynamic effects of delayed cord clamping are receiving increasing scrutiny. While there is debate as to the safety and feasibility of delayed cord clamping, particularly among infants warranting immediate resuscitation, well-designed animal studies have demonstrated improved mean arterial pressure and cardiac output in near-term asphyxiated lambs that received delayed cord clamping [18]. In addition, a recent randomized controlled trial by Katheria et al. involving 60 neonates, which examined the feasibility of performing one versus five minutes of delayed cord clamping in term neonates deemed at risk for resuscitation, demonstrated five minutes of delayed cord clamping appeared safe without compromising resuscitative efforts [19]. Furthermore, the neonates that received five minutes of delayed cord clamping also had better mean blood pressures and cerebral oxygenation compared to the group that only received one minute of delayed cord clamping. Similar concerns in preterm neonates were addressed in a recent pilot study by Winter et al. involving 29 neonates born between 24 0/7 and 32 6/7 weeks’ gestation, which demonstrated that immediate ventilation and resuscitation during the period of delayed cord clamping for 90 s was both feasible and safe [20]. The authors were able to successfully perform their normal resuscitation protocol in all neonates enrolled with the umbilical cord still intact; variables such as heart rate, Apgar scores, cord blood gas values, and admission temperatures were comparable to their institutional norms. Given the likely benefits of delayed cord clamping in promoting a more stable birth transition (arguably even in neonates requiring immediate resuscitation), health care providers are left with some unanswered questions: 1) Have we unknowingly “interfered” with this period of transitional physiology by clamping and cutting the cord too early? 2) Do we need to re-evaluate what are considered normal biophysical and laboratory markers in this population?

Most of the available vital signs and laboratory reference ranges related to newborns were derived from neonatal reference group populations who likely had early cord clamping since this was the standard practice for the past century. Therefore, whether or not we can assume these neonates represent healthy “normal” neonates, who encompass ~ 95% of the total neonatal population, requires further study. Caution with how we define what is *normal* is warranted as this process has shortcomings; linking outcomes to reference ranges is problematic given the diversity of neonatal phenotypes and epigenetic factors that are often understudied, unavailable, or underappreciated.

One such *normal* reference range example involves hemoglobin and hematocrit reference ranges in preterm neonates. The reference ranges, which are designed to try to approximate normal values, are largely compiled from tests performed on neonates that are assumed to either have minimal or irrelevant pathology that may affect the test. The majority of established neonatal reference ranges for hematocrit and hemoglobin concentration were based on small population studies (often < 100 patients) and inaccurate methodology. In comparison to these established ranges, more recent reference ranges derived using modern hematology analyzers should be considered, such as values determined by Jopling et al., who studied a large cohort of neonates between 22 to 42 weeks’ gestation and reported hematocrit (*N* = 41,957 patients) and hemoglobin (*N* = 39,559 patients) ranges during the first 28 days after birth [21]. Interestingly, the authors acknowledge their finding of a “lack of postbirth increase” in hematocrit in infants < 35 weeks’ gestation as largely attributable to the “practice of rapidly clamping the umbilical cord.” This lack of postbirth increase is concerning, given the vast amount of evidence demonstrating that neonates who receive delayed cord clamping have a significant increase in circulating blood volume and hemoglobin. Knowledge of these hematocrit discrepancies between cord clamping practices should make us re-consider how we reach conclusions regarding *normal* values, since a better regard for context (e.g., lack of a placental-to-neonatal transfusion) may improve how we understand newborn physiology and react to data. This same notion can be applied to other typically monitored, but often debated, parameters, including admission temperatures and goal oxygen saturations in neonates transitioning after birth. A recent study by Smit et al. compared pulse oximetry and heart rate changes in healthy neonates that received delayed cord clamping and immediate skin-to-skin contact versus internationally defined reference ranges that typically are formulated from neonates that receive immediate cord clamping [22]. They discovered that neonates who received delayed cord clamping had higher SpO2 values in the first few minutes of age and lower median heart rates across all time points. These significant changes in the first few minutes after birth reflect differences at the 10th percentile of currently accepted ranges, again calling into question the ranges that we commonly refer to as “normal.” Our understanding of the status quo, similar to past and current views of target oxygen saturation goals, may be skewed by our potentially indifferent approach of early transition.

## Re-classify: Delayed cord clamping is the standard, early is the experimental

A recent systematic review and meta-analysis by Fogarty et al., which included 18 randomized controlled trials (2834 infants born < 37 weeks’ gestation), demonstrated that delayed cord clamping reduced hospital mortality compared to early cord clamping [8]. The authors acknowledge that “delayed clamping is more closely aligned to natural birth,” but “for the purposes of analysis, delayed cord clamping was regarded as the experimental treatment, as in previous systematic reviews.” To make the systematic analysis consistent, treating delayed cord clamping similar to previous studies was appropriate. However, continuing to portray delayed cord clamping as the unconventional intervention in the article’s discussion reinforces the idea that delayed cord clamping is the unproven intervention and potentially distorts the correct conclusions, which should be reached when early cord clamping is treated as the experimental intervention.

The American Academy of Pediatrics, the American College of Obstetrics and Gynecology, and International Liaison Committee on Resuscitation all support the practice of delayed cord clamping among preterm births [3–5]. However, despite this evidence-based endorsement, categorizing delayed cord clamping, and not early cord clamping, as the unconventional intervention perpetuates the mindset that early cord clamping is without risk, a perspective that is false. Thus, we propose the better wording for the systematic review’s primary finding is that there is strong indication that early cord clamping results in an increased all-cause mortality before discharge from hospital (risk ratio 1.5, 95% Confidence Interval 1.1–1.9; *P* = 0.006).

Extending this rewording further would result in the following revised statements:

*For the infant, early cord clamping appears to provide no benefit, but there was a trend towards lower Apgar scores and a greater need for resuscitation and intubation at delivery. There was a trend towards an increase in the key neonatal morbidities of severe intraventricular hemorrhage, severe retinopathy of prematurity, chronic lung disease, necrotizing enterocolitis, and late onset sepsis.*

These reworded examples demonstrate how the conclusions are significantly altered when early cord clamping is the experimental intervention. Given that the systemic review findings by Fogarty et al. align with recommendations from governing bodies, we recommend that delayed cord clamping be viewed as the standard of care and early cord clamping as the experimental intervention in all future studies. Continuing to treat early cord clamping as the standard intervention in studies and reviews perpetuates a misleading message that potentially promotes a casual attitude toward placental transfusion practices, which may have a significant negative impact on neonatal outcomes.

Evidence has shown that natural air, an FiO_2_ of 0.21, can be used to safely resuscitate term neonates [23, 24] and subsequent evidence using air to resuscitate mature hypoxic neonates demonstrated a significantly reduced risk of death [25]. This evidence has changed our guidelines and practice of immediately increasing oxygen in the delivery room [26–29]. In a similar fashion, but in preterm neonates, delayed cord clamping has been shown to be safe [7] and now also reduce mortality [8]. While guidelines have also changed to recommend delayed cord clamping, this practice change has been met with less acceptance than recommendations on modifying oxygen use or stopping narcan or sodium bicarbonate use during newborn resuscitations, despite substantially more evidence to support a practice change advocating delayed cord clamping than some of these other endorsements. Previously, providers concerned about hypoxia in the nonvigorous newborn were quick to increase supplemental oxygen to achieve preductal SpO2 values that we now target to achieve at 10 min of age (85% to 95%) [3]. After the revised preductal SpO2 target value guidelines were introduced, a cultural shift occurred over time, where providers became more comfortable in regards to preductal SpO2 values that in the past they may have considered troublesome and been inclined to increase supplemental oxygen. Similar to any recommended change in established practice guidelines, such as supplemental oxygen use for newborn resuscitation, modifying the timing of umbilical cord clamping will require providers to become accustomed to new management practices intended to benefit newborns, especially premature newborns, before a cultural practice shift occurs.

Many providers remain reluctant to “delay” resuscitation in a nonvigorous newborn, and therefore opt for early cord clamping to rescue the baby. However, as demonstrated by our previously misguided perceptions that newborns needed immediate high SpO2 values, modifying our practice to choose delayed cord clamping (i.e., 30–60 s in a preterm neonate) over immediate cord clamping should be viewed as an active treatment strategy instead of an a delay in resuscitation. To deny premature newborns, especially at the extremes of prematurity, the potential benefits of delayed cord clamping in order to “resuscitate” them faster disregards the role of placental-to-newborn transfusion as part of the natural birth transition and also ignores the harmful risks of early cord clamping. While more studies are needed to clarify the optimal approach to placental-to-newborn in nonvigorous newborns, waiting for newborns to breathe spontaneously before clamping the cord may lead to better outcomes. This point is suggested by an observational study by Ersdal et al. performed in Tanzania that demonstrated healthy, low birth weight (< 2500 g) neonates who were not spontaneously breathing after birth had an increased chance of dying if their cord was clamped before or immediately after they began to spontaneously breathe [30]. In these neonates, every 10-s delay in clamping after breathing decreased death or the need for hospital admission by 20%.

## Conclusion

To provide the benefits of placental-to-newborn transfusion practice in a hospital setting, we urge that institutions broadly apply delayed cord clamping to all newborns, except within a well-defined cohort of mother-infant dyads (see Additional file 1: Figure S1). Acceptance of this practice warrants engagement of a dedicated leadership team to educate and motivate key stakeholders, find methods to measure and encourage staff compliance, and determine ways to track outcome data of infants who underwent delayed cord clamping [31]. While more studies are needed to address unanswered questions pertaining to placental-to-newborn transfusion strategies, important evidence-based benefits, including the analysis by Fogarty et al. demonstrating decreased mortality, support the practice of delayed cord clamping in premature neonates. Since in medicine, *what is in a name* may have grave implications, future studies in preterm neonates should consider changing early cord clamping to the experimental approach and making delayed cord clamping for 30–60 s the standard practice.

## Additional file

Additional file 1: **Figure S1.** Delayed cord clamping algorithm flowsheet. (JPEG 503 kb)

## Abbreviations

Hb: Hemoglobin
SpO_2_: Peripheral capillary oxygen saturation
