# Supplementary figures and images for: Revert to the original: time to re-establish delayed umbilical cord clamping as the standard approach for preterm neonates
Source: Matern Health Neonatol Perinatol. 2018 Jul 4;4:13. doi: 10.1186/s40748-018-0081-5 (PMC6030773; doi:10.1186/s40748-018-0081-5)

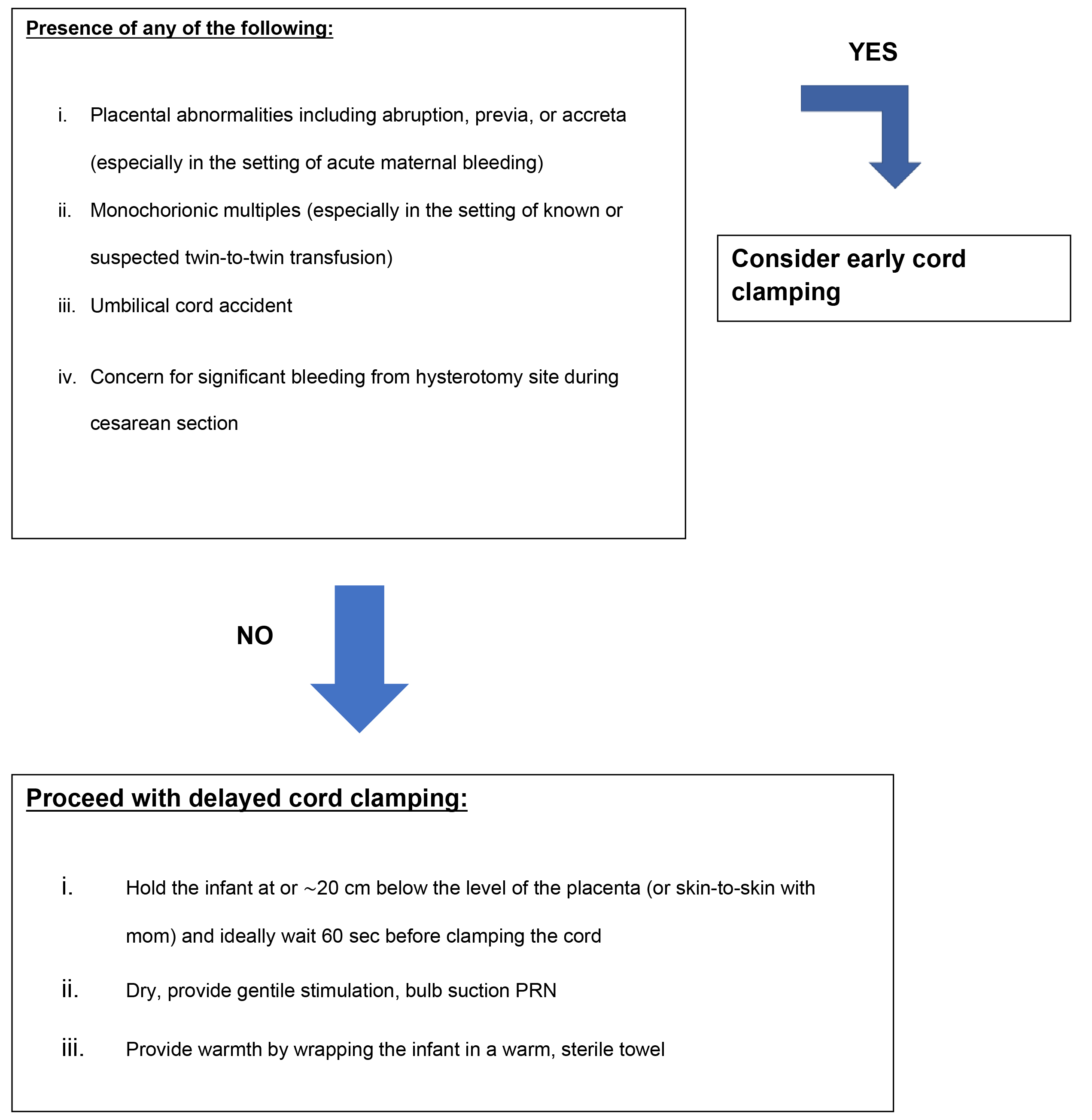

Supplement: Supplementary file 1 — Figure S1. Delayed cord clamping algorithm flowsheet. (JPEG 503 kb) [file 40748_2018_81_MOESM1_ESM.jpg]
